# Approach to the Patient With Suspected Hypotonic Polyuria

**DOI:** 10.1210/clinem/dgae565

**Published:** 2024-08-16

**Authors:** John Newell-Price, Juliana Beaudette Drummond, Mark Gurnell, Miles Levy, Ann McCormack, Deborah Cooper, John Wass, Mirjam Christ-Crain, Joseph G Verbalis

**Affiliations:** School of Medicine and Population Health, University of Sheffield, Sheffield S10 2RX, UK; Department of Internal Medicine, School of Medicine, Federal University of Minas Gerais, Belo Horizonte MG - CEP 31270-901, Brazil; Institute of Metabolic Science & Department of Medicine, University of Cambridge & Addenbrooke's Hospital, Cambridge Biomedical Campus, Cambridge CB2 0QQ, UK; Department of Endocrinology, University Hospitals of Leicester NHS Trust, Leicester LE3 9QP, UK; Department of Endocrinology, St Vincent's Hospital, Sydney, NSW 2010, Australia; Hormones and Cancer Group, Garvan Institute of Medical Research, Sydney, NSW 2010, Australia; St. Vincent's Clinical School, University of New South Wales, Sydney, NSW 2052, Australia; The Pituitary Foundation, Bristol BS2 8PE, UK; Department of Endocrinology, University of Oxford, Churchill Hospital, Oxford OX3 7LE, UK; Department of Endocrinology, University Hospital and University of Basel, CH-4031 Basel, Switzerland; Georgetown-Howard Universities Center for Clinical and Translational Science, Georgetown University, Washington, DC 20007, USA

**Keywords:** arginine vasopressin, deficiency, resistance, polydipsia, hypotonic, polyuria, diabetes insipidus, osmolality, systemized nomenclature of medicine (SNOMED)

## Abstract

Investigation and management of hypotonic polyuria is a common challenge in clinical endocrinology. The 3 main causes, recently renamed to *arginine vasopressin deficiency* (AVP-D, formerly *central diabetes insipidus*), *arginine vasopressin resistance* (AVP-R, formerly *nephrogenic diabetes insipidus*), and *primary polydipsia* (PP) require accurate diagnosis, as management differs for each. This new nomenclature more accurately reflects pathophysiology and has now been adopted by the Systemized Nomenclature of Medicine (SNOMED). Advances in diagnosis over the last few years have centered around the use of copeptin measurement. Here, we use 3 patient case histories to highlight the use of this approach, and to demonstrate how it can succeed where other approaches, such as the water deprivation test, sometimes fail. We discuss the overall approach to each type of patient and the strengths and limitations of diagnostic strategies, illustrating the use of the new nomenclature.

It is common for endocrinologists to receive referred patients who have polyuria and polydipsia that need investigation. When initial investigations reveal the urine to be hypotonic, differentiating between the 3 main causes is essential, as the management differs for each (1). Over the past 2 years, due to concerns about patient safety and in response to patient feedback, the causes of hypotonic polyuria have been renamed as *arginine vasopressin deficiency* (AVP-D, formerly *central diabetes insipidus*), *arginine vasopressin resistance* (AVP-R, formerly *nephrogenic diabetes insipidus*), while the name of the third cause is retained as *primary polydipsia* (PP), in which excess fluid intake suppresses AVP (2) secretion. In a survey of more than one thousand patients with AVP-D, the large majority had experienced confusion of their condition by health care workers with *diabetes mellitus*, resulting in delays in availability of desmopressin and unnecessary capillary glucose monitoring when hospitalized, and 85% favored changing the name (3). The new nomenclature more accurately reflects pathophysiology and clearly identifies the patients’ condition as different from diabetes mellitus, and now has been adopted by the Systemized Nomenclature of Medicine (SNOMED) taxonomy hierarchy codes for electronic patient records (4). In parallel, the last several years have seen improvements in the diagnostic tools used for the investigation of hypotonic polyuria, with a particular emphasis on the use of measurement of plasma copeptin. This Approach to the Patient review presents the use of this copeptin-based approach in 3 specific cases, and places each in the context of the revised nomenclature. We then describe the overall approach to the patient presenting with hypotonic polyuria, itemize the causes, and discuss when and how testing should be performed (Fig. 1), highlighting specific areas that frequently cause diagnostic and management difficulties.

**Figure 1.**
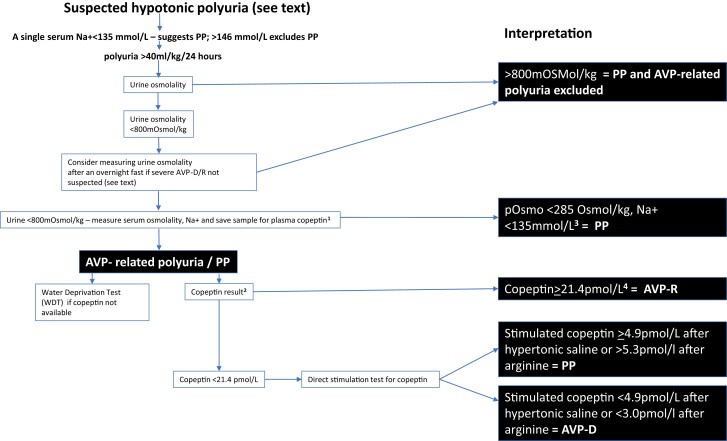
Flow diagram for the approach to the patient with hypotonic polyuria. Abbreviations: AVP-D, arginine vasopressin deficiency; AVP-R, arginine vasopressin resistance; PP, primary polydipsia. ^1^AVP measurement validated in some countries; ^2^ Water deprivation test an alternative; ^3^Actual cutoff depends on reference range of serum Na+; ^4^Nonosmotic stimulation—for example—acutely unwell/syncope may cause an elevated level. Interpretation of water deprivation test: If copeptin is not available, the indirect water deprivation test can be performed as described by Miller et al (5). Usually, the concentration of urine over a 16-hour period of fluid restriction is assessed, followed by interpretation of the ability to concentrate urine in response to the synthetic V2 receptor-specific AVP analogue, desmopressin at the end of the test. In patients where the urinary osmolality stays below 300 mOsm/kg during fluid deprivation but increases over 50% upon desmopressin administration, the diagnosis of complete AVP-D can be made. Patients with a persistent low urinary osmolality and no response to desmopressin are diagnosed with AVP-R. Urinary concentration in patients with partial AVP and primary polydipsia is expected to increase to levels above 300 mOsm/kg, whilst remaining below 800 mOsm/kg, with further increases in osmolality seen upon desmopressin injection of more than 9% in partial AVP patients and less than 9% in primary polydipsic patients (5).

## Case 1

### Case 1 Presentation and Assessment

A 77-year-old male patient was referred for further evaluation of possible AVP-D. The patient reported onset of polyuria and polydipsia following a viral infection that was thought to be COVID-19, but this was never confirmed. The symptoms of polyuria and polydipsia persisted but slowly decreased in intensity over the next 2 years. The patient's symptom diary recorded total urine volumes between 2 to 2.9 liters (L)/24 hours, nocturia 1 to 2 times/night and daytime urinary frequency, but without large volumes of urine with each void. He estimated his fluid intake to be approximately 2.0 L/day. His thirst was described as moderate (4/10) but was usually easily relieved with small sips of water. In his past medical history, he had received lithium for more than 30 years for bipolar disorder. When he reported polyuria and polydipsia, his psychiatrist stopped the lithium approximately 1 year ago, without much effect on his polyuria. He also had a past history of bladder outlet obstruction, bladder cancer, and benign prostatic hypertrophy treated with tamsulosin 0.4 mg twice a day. His other medications included quetiapine 300 mg/d and atorvastatin 20 mg/d. Prior to referral he had undergone an 8-hour overnight fluid deprivation, following which his biochemical testing showed serum sodium 144 mmol/L (133-146), estimated glomerular filtration rate (eGFR) = 54 mL/min, plasma osmolality 301 mOsm/kg, and urine osmolality 390 mOsm/kg, but desmopressin was not administered at the end of the overnight fluid deprivation. Other biochemistry was normal including glycated hemoglobin (HbA1c) = 5.9% (4.0-5.6%), free thyroxine (free T4) = 1.4 ng/dL (0.8-1.5) (SI: 18 pmol/L [10.3-19.8 pmol/L]), thyrotropin (TSH) = 1.42 mIU/L (0.5-5.0), serum calcium 2.34 mmol/L (2.2-2.7), LiCl < 0.1 mmol/L. These results were thought by the referring endocrinologist to be most consistent with AVP-D.

On physical examination, the patient was clinically euvolemic with blood pressure = 153/88 mmHg, weight = 80.1 kg, and body mass index = 28 kg/m^2^. Evaluation consisted of a single fasting early morning serum copeptin level, which was elevated at 22.8 pmol/L, confirming a diagnosis of AVP-R (Fig. 1). In retrospect, a call to the referring endocrinologist's office revealed that a copeptin level was 64.8 pmol/L at the end of the overnight fluid deprivation test but had not been recorded in the referral due to a delay in the assay results being returned by the laboratory.

### Case 1 Discussion

Although the patient did not meet criteria for a diagnosis of polyuria (Fig. 1), 2 factors influenced the decision for further investigation: (i) the patient's chronic kidney disease likely limited the total urine output; and (ii) the referring endocrinologist was concerned about the possibility of AVP-D, in which case the patient would respond to treatment with desmopressin. The definition of hypotonic polyuria is discussed in the subsequent section on “Clinical Suspicion,” but this case illustrates that clinical judgment and experience should be considered in the identification of patients who might benefit from further evaluation. An elevated plasma AVP level has long been a diagnostic feature of AVP-R. Variability in immunochemical assays for AVP and the inherent challenges in measuring this labile peptide, has, however, precluded widespread adoption of formal cutoff values for making this diagnosis. The development of the BRAHMS copeptin assay has enabled formal criteria to be developed, and a cutoff of 21.4 pmol/L in a baseline plasma sample has 100% specificity and sensitivity for diagnosis of AVP-R, without the need for further testing (6, 7). This case was confusing to the referring endocrinologist because the patient had discontinued lithium therapy more than a year ago, and the intercurrent COVID infection raised a question as to whether there could possibly be central nervous system (CNS) effects and possible AVP-D. Polyuria due to impaired urinary concentrating ability occurs in up to 20% of patients treated with chronic lithium therapy; an additional 30% have a subclinical impairment in concentrating ability. These effects are mediated by lithium entry into the principal cells in the collecting tubule via the epithelial sodium channel (ENaC), where lithium inhibits signaling pathways that involve glycogen synthase kinase type 3 beta (GSK3beta), resulting in dysfunction of the aquaporin-2 water channels (8). Although most cases of lithium-induced AVP-R resolve after discontinuation of lithium therapy, AVP-R often becomes irreversible after many years of chronic use. Although the mechanism is incompletely understood, it is thought to be the result of a chronic tubulointerstitial nephropathy. The markedly increased copeptin level after an overnight fluid deprivation shows that AVP secretion can increase further in response to osmotic or volume stimuli, but establishing this is not necessary for the diagnosis of AVP-R. One potential confounder in this case is the patient's underlying chronic kidney disease, which has been shown to elevate serum copeptin levels. This effect is, however, most marked with eGFR levels < 50 mL/min, which was not the case here. In addition, the patient's past urological history and related medication may have caused diagnostic confusion, and although the HbA1c was not entirely normal, and was consistent with prediabetes, it did not represent a degree of glucose dysregulation that would be associated with polyuria from glucosuria. Treatment of AVP-R includes reduced protein and NaCl intake, hydrochlorothiazide, amiloride, and nonsteroidal anti-inflammatory drugs (1). In this case, amiloride was not indicated because the patient was no longer taking LiCl, and other pharmacological agents were avoided to prevent accelerated progression of CKD. Modest protein and NaCl restriction were recommended to reduce urine output and limit the patient's urinary frequency and nocturia.

## Case 2

### Case 2 Presentation and Assessment

A 29-year-old male patient presented with a 12-year history of polyuria and polydipsia. The onset of symptoms had been gradual, and he described drinking around 12 L/day and polyuria with around 16 episodes of voiding during the day but only 1 or 2 during the night. Family history was negative for polyuria and polydipsia. In his past medical history, he had an obsessive-compulsive disorder and attention-deficit/hyperactivity disorder. Medication included duloxetine 60 mg/d and Ritalin 10 mg twice a day. Prior to referral, an indirect water deprivation test had been nondiagnostic: maximum urine osmolality of 543 mOsm/kg after 8 hours of water deprivation with serum sodium 142 mmol/L and a plasma osmolality 297 mOsm/kg. A further 41% maximum increase in urine osmolality (765 mOsm/kg) was observed 90 minutes following administration of desmopressin 2 mcg intravenously.

After referral, a 24-hour urine collection revealed a urinary volume of 15.8 L (148 mL/kg/24 hours) and a urine osmolality of 113 mOsm/kg, confirming hypotonic polyuria. Baseline serum sodium was 140 mmol/L, with normal renal function, serum glucose, and calcium. Pituitary magnetic resonance imaging (MRI) was normal with a posterior pituitary bright spot visible. His baseline plasma copeptin level of 3.6 pmol/L, excluding AVP-R (Fig. 1).

In order to better differentiate between primary PP and partial AVP-D, the patient underwent a hypertonic saline stimulation test. Serum sodium levels reached 150 mmol/L after a 3-hour infusion of NaCl 3% (0.15 mL/kg/min). The procedure was well-tolerated, and the patient reported severe thirst (10/10 on a visual analogue scale) at the end of the test. Hypertonic saline-stimulated copeptin level was 29.2 pmol/L, confirming the diagnosis of PP.

Behavioral therapy was recommended. On a follow-up visit, after 3 months, the patient reported partial improvement as he was able to slightly reduce water intake with a reduction of voiding to around 10 episodes/24 hours and with a urinary volume decrease of approximately 30% (95 mL/kg/24 hours).

### Case 2 Discussion

The differentiation between PP and partial AVP-D is often challenging. Features in this patient's clinical history that favor the diagnosis of PP include the gradual onset of symptoms as well as less polyuria at night. Even though psychiatric disease has been classically associated with PP, a recent international patient survey showed a high prevalence of psychological comorbidities in patients with AVP-D (9). A 24-hour urine collection is essential to establish the diagnosis of PP characterized by urinary volume above 50 mL/kg/24 hours and urine osmolality below 600 mOsm/kg; however, the amount of polyuria or the urine osmolality per se do not aid in the distinction between PP and partial AVP-D. Most patients with PP will present with normal serum sodium levels, while hypotonic hyponatremia is indicative of PP. The water deprivation test is frequently nondiagnostic in patients with PP and partial AVP-D since water deprivation is commonly interrupted before serum sodium concentration or plasma osmolality increases sufficiently to stimulate maximal AVP secretion, in which case desmopressin administration will cause a further increase in urine osmolality, even in patients with PP (10), as illustrated by this case. The hypertonic saline-stimulated copeptin is the gold standard test to differentiate PP from AVP-D and a copeptin level > 4.9 pmol/L is diagnostic of PP with an accuracy above 95% (7, 11). While hypertonic saline infusion is usually well-tolerated, common side effects include headache, nausea, and thirst, but these are most often mild. The test should be performed under medical supervision at experienced centers, as it requires close monitoring of sodium levels to ascertain a diagnostically meaningful increase in plasma sodium within the hyperosmotic range while preventing a marked increase.

## Case 3

### Case 3 Presentation and Assessment

A 50-year-old man reported an 18-month history of increasing tiredness and lethargy with reduced exercise tolerance. More recently, he had developed significant thirst and polyuria (4-5 L per 24 hours) with associated sleep disturbance (nocturia 4-6 times per night). He described no other lower urinary tract symptoms. Initial investigation excluded diabetes mellitus.

Careful questioning revealed that he had undergone excision biopsy of a solitary cervical lymph node 8 years earlier, with histology demonstrating numerous non-caseating granulomas. Further investigation had excluded systemic involvement, and a diagnosis of localized sarcoidosis was made. No treatment was recommended.

At re-presentation, although physical examination was unremarkable, the patient observed he had reduced muscle mass and central adiposity when compared with previous examinations.

Assessment of anterior pituitary function revealed central hypogonadism, luteinizing hormone = 0.4 mIU/mL (1.5-6.3) (SI 0.4 IU/L [1.5-6.3]), total testosterone = 23 ng/dL (194-900) (SI: 0.8 nmol/L [6.7-31.3]), with mild hyperprolactinemia, prolactin = 33.5 ng/mL (2.1-17.6) (SI: 713 mU/L [45-375]). His 9 Am cortisol level was 16.3 mcg/dL (5-25) (SI: 450 nmol/L [137-690)], free T4 1.08 ng/dL (0.8-1.5) (SI: 13.9 pmol/L [10.3-19.8 pmol/L]), and TSH 0.56 mU/L (0.35-5.5). Early morning serum sodium was 146 mmol/L (133-146), plasma osmolality 292 mOsm/kg and urine osmolality 351 mOsm/kg. During an indirect water deprivation test, urine osmolality reached a peak of 524 mOsm/kg after desmopressin administration.

**Figure 2.**
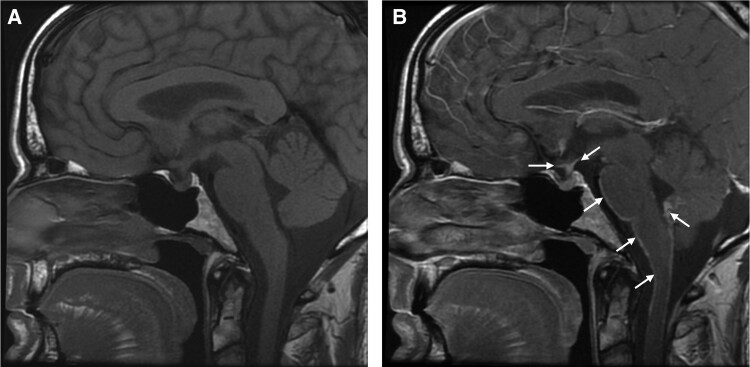
Case 3: T1-weighted MRI pre- (A) and post- (B) gadolinium. The posterior pituitary bright spot is absent on the noncontrast imaging. Following gadolinium administration, there is abnormal enhancement along the floor of the third ventricle (upper two arrows), together with widespread leptomeningeal enhancement (lower four arrows).

Pre-contrast MRI of the sella region demonstrated absence of the normal posterior pituitary bright spot. Following gadolinium injection, abnormal enhancement was observed along the floor of the third ventricle and inferior to the optic chiasm with further leptomeningeal enhancement over the surface of the brainstem, cerebellum, and cervical cord (Fig. 2).

A diagnosis of neurosarcoidosis was made and treatment commenced with prednisolone 60 mg daily. In view of continued thirst and polyuria (with normal plasma glucose), once-daily desmopressin was started with measurement of serum sodium at 24 and 72 hours, and after 1 and 2 weeks, following which the patient reported a marked improvement in his symptoms. Subsequent hypertonic saline-stimulated copeptin measurement (with desmopressin omitted on the day of the test) was consistent with AVP-D (serum copeptin 2.3 pmol/L when serum sodium > 150 mmol/L) (Fig. 1).

### Case 3 Discussion

With the clinical history, previous biopsy findings, and imaging abnormalities, the likelihood that this patient had AVP-D rather than PP was high. It was for this reason that despite the inconclusive water deprivation test he was commenced on desmopressin, with close observation. Subsequent hypertonic-stimulated copeptin levels then confirmed AVP-D rather than PP. MRI imaging of the hypothalamic-pituitary region is useful to evaluate for the presence of tumors/infiltrative disease as well for the presence of the posterior pituitary bright spot. Lack of a posterior pituitary bright spot (Fig. 2) is, however, not uniform in patients with AVP-D and may occur in PP: in a recent multicenter international study, the pituitary bright spot was absent in 68% of patients with AVP-D and in 14% of patients with PP, highlighting that MRI results need to be interpreted in the clinical context (7, 11). It is important to note that cortisol deficiency, while not a feature of this case, may mask AVP-D which then becomes manifest with glucocorticoid replacement (1).

## Overall Approach to the Patient

### Clinical Suspicion

Patients who have symptoms of polyuria, nocturia, or polydipsia are candidates for further evaluation of a possible arginine vasopressin-related polyuria (AVP-D or AVP-R). However, before embarking on more complex pathways of investigation, it is important to exclude common causes of polyuria and polydipsia, including hypokalemia and hypercalcemia. Certain scenarios are suggestive of a solute diuresis, for example uncontrolled hyperglycemia, high protein intake from parenteral or enteral feeding, tissue catabolism from high-dose glucocorticoids, resolving kidney injury including relief of urinary obstruction, use of diuretics and SGLT-2 inhibitors, administration of mannitol or high volumes of isotonic NaCl. The presence of nocturia without daytime polyuria suggests nocturnal polyuria (production of 20%-33% of total urine volume during sleep). In the absence of such obvious causes of symptomatic polyuria, possible AVP-related disorders should be evaluated. The first step is measurement of plasma osmolality and/or serum sodium concentration. Although these are within normal ranges in most patients with polyuria due to the ability of thirst and renal water excretion to maintain osmotic homeostasis, hypernatremia strongly suggests AVP-D or AVP-R, and hyponatremia strongly suggests PP as the most likely causes of polyuria (1). If the plasma osmolality and serum sodium are normal, the next step is confirming the presence of hypotonic polyuria. Because patients’ estimations of both fluid intake and urine output are not always reliable, this should be assessed via measurement of a 24-hour urine (1). It is notable that there is no universally accepted definition of polyuria. Past studies have used an absolute urine volume (eg, ≥ 3 L/24 hours in adults and ≥ 2 L/m^2^/24 hours in children), and others have used a weight-based urine volume (eg, > 40 mL/kg/24 hours or > 50 mL/kg/24 hours). Consequently, clinical judgment and experience should be used to identify patients requiring further evaluation, but the least stringent criteria would be either a urine volume ≥ 3 L/24 hours in adults and ≥ 2 L/m^2^/24 hours in children, or > 40 mL/kg/24 hours. If these minimal criteria are not met, the patient should undergo urological evaluation for causes of increased urine frequency or nocturnal polyuria. Once polyuria is established, the second step is to confirm hypotonicity via measurement of osmolality in the 24 hours urine sample. A urine osmolality ≤ 300 mOsm/kg H_2_O confirms a hypotonic urine, and a urine osmolality ≥ 800 mOsm/kg H_2_O indicates a solute diuresis (due to glucose, sodium, urea, or administered diuretics such as mannitol). Urine osmolalities between 300 and 800 mOsm/kg H_2_O can be seen with partial AVP-D or AVP-R, so in such cases further evaluation is necessary. Once hypotonic polyuria is confirmed, additional testing is necessary to identify the cause (Table 1).

**Table 1.** Etiology of polyuria polydipsia syndromes

Basic defect	Acquired causes	Hereditary/congenital causes
**Arginine vasopressin deficiency (AVP-D/formerly known as central diabetes insipidus)**		
Deficiency in AVP synthesis and secretion	Trauma (transsphenoidal surgery*^a^*, intracranial surgery, deceleration injury) Neoplasia (craniopharyngioma, germinoma, meningioma, metastasis to the pituitary and/or hypothalamus) Vascular (cerebral or hypothalamic hemorrhage and infarction, anterior communicating artery aneurysm or ligation, Sheehan syndrome, sickle cell disease) Infectious (meningitis, encephalitis, tuberculosis, pituitary or hypothalamic abscess) Granulomatous (sarcoidosis, Langerhans cell histiocytosis, Erdheim-Chester disease, granulomatosis with polyangiitis) Inflammatory or autoimmune (lymphocytic infundibulo-neurohypophysitis, IgG4 neurohypophysitis, anti-vasopressin neuron antibodies, Guillain-Barré syndrome) Osmoreceptor dysfunction (adipsic AVP-D) Drug or toxin-exposure (snake venom, tetrodotoxin, phenytoin, ethyl alcohol) Others (hydrocephalus, ventricular or suprasellar cyst, degenerative diseases) Idiopathic	Autosomal dominant: *AVP* gene mutations Autosomal recessive: type a and b: *AVP* gene mutations; type c: *WFS1* gene mutation—Wolfram (DIDMOAD) syndrome; type d: *PCSK1* gene mutation (AVP-D + extreme obesity) X-linked recessive: gene unknown Septo-optic dysplasia Schinzel-Giedion syndrome Culler-Jones syndrome Alstrom syndrome Hartsfield syndrome Webb-Dattani (WEDAS) syndrome
**Arginine vasopressin resistance (AVP-R/formerly known as nephrogenic diabetes insipidus)**		
Reduced renal sensitivity to antidiuretic effect of physiological AVP levels	Medications (lithium, demeclocycline, cisplatin, aminoglycosides, amphotericin B, methoxyflurane, sevoflurane, etc.) Metabolic (hypercalcemia or hypokalemia) Infiltration associated with systemic disease (amyloidosis, sarcoidosis, Sjogren syndrome, hemochromatosis, multiple myeloma, etc.) Vascular (renal infarction, sickle cell disease) Mechanical (polycystic kidney disease and urethral obstruction)	X-linked *AVPR2* mutations (∼90% of the cases) Autosomal recessive or dominant *AQP2* gene mutations Polyhydramnios, megalencephaly and symptomatic epilepsy (PMSE) syndrome Inherited renal syndromes (nephropathic cystinosis, Bartter syndrome, nephronophthisis, etc.)
**Primary polydipsia**		
Excessive fluid intake at a diminished set point	Dipsogenic (downward resetting of the thirst threshold): idiopathic or similar intracranial lesions as with AVP-D Health enthusiasts Compulsive water drinking Psychosis-intermittent hyponatremia polydipsia (PIP) syndrome Medication-induced: anticholinergics, phenothiazines	NA
**Pregnancy-induced/gestational arginine vasopressin-related polyuria(/formerly known as gestational diabetes insipidus)**		
Increased enzymatic metabolism of circulating AVP	Pregnancy—vasopressinase activity by placenta	NA

NA: not applicable

^*a*^Anterior pituitary adenoma very rarely causes AVP-D before pituitary surgery.

### Rationale for Use of Copeptin as Diagnostic Approach

The precursor peptide of AVP consists of the AVP moiety, neurophysin-2, and a 39–amino acid glycosylated peptide with a leucine-rich core segment, termed copeptin at the C-terminal end. This precursor is synthesized in magnocellular neurons located in 2 discrete areas of the hypothalamus, the supraoptic and paraventricular nuclei, and during axonal transport to the posterior pituitary it is cleaved to form AVP and copeptin, and both are released into the circulation in equimolar amounts (12, 13). AVP is the main regulatory hormone for water homeostasis. During normal day-to-day living, AVP release is mainly stimulated by an increase in plasma osmolality and induces water reabsorption via AVP V2 receptors in the kidneys (14). Nonosmotic release may also occur during significant hypovolemia and high levels of physical stress, trauma, nausea, or illness. Inadequate secretion, or more usually deficient synthesis, of AVP in the hypothalamic neurohypophyseal system results in AVP-D. AVP measurement in response to osmotic stimulation has therefore long been recommended in the diagnostic evaluation of patients with polyuria polydipsia (15) (see subsequent section on “Use of Water Deprivation Tests”).

AVP is, however, difficult to measure due to complex pre-analytical requirements and lack of readily available assays with a quick turnaround for the result. Moreover, a large amount of AVP in the circulation is bound to platelets, resulting in underestimation of actual AVP concentration, while incomplete removal of platelets from plasma or prolonged storage of unprocessed blood leads to falsely elevated AVP measurements (16-18). In addition, detection of AVP is also hampered by its short half-life in vivo of less than 30 minutes and its instability in isolated plasma, even when stored at −20 °C. For these reasons, direct measurement of AVP is not used in clinical practice in most countries, although there are exceptions—for example Japan—where validated assays are available in the differential diagnosis of polyuria polydipsia syndrome (19).

Copeptin responds as rapidly as AVP to osmotic and nonosmotic stimuli, which is explained by its equimolar production and secretion together with AVP. A direct comparison between copeptin and AVP release in relationship to serum osmolality showed a stronger correlation of plasma osmolality with copeptin than with AVP and a very strong correlation between both peptides (20). A study directly comparing the kinetics and half-life of copeptin and AVP showed similar kinetics of copeptin secretion in response to increases in osmotic pressure to AVP. The half-life of copeptin is, however, around 2 times higher than the half-life of AVP, reflecting the differing volume of distribution and metabolic clearance rates of the 2 peptides (21).

Due to its high stability ex vivo and simple and robust measurement, copeptin offers an alternative method to indirectly assess the release of AVP. There are several copeptin assays available. The 2 assays with sufficient technical reliability and clinical data to justify their routine clinical use are the original sandwich immunoluminometric assay (LIA) (22) and its automated immunofluorescent successor (on the KRYPTOR platform) [both Thermo Fisher Scientific BRAHMS GmbH Neuendorfstr. 25 16761 Hennigsdorf Germany]. A recent study showed that copeptin measured by the KRYPTOR and LIA assays showed a good correlation over a wide range of copeptin concentrations (from very low to very high levels). In contrast, copeptin measured by an enzyme-linked immunosorbent assay (ELISA) method correlated only poorly with both the KRYPTOR and LIA measured copeptin concentrations (23).

With the measurement of copeptin, the diagnostic evaluation of AVP-D greatly improved. Over the last few years, several copeptin-based tests have shown their superiority to the indirect water deprivation test (7, 24).

### Copeptin Stimulation Tests in the Differential Diagnosis of Polyuria Polydipsia Syndrome

Baseline unstimulated copeptin levels can be used to diagnose AVP-R where patients usually present with a copeptin level ≥ 21.4 pmol/L (6, 10) (Fig. 1). However, baseline copeptin levels show a large overlap in patients with AVP-D and PP (6) and to reliably diagnose AVP-D, a stimulation test for copeptin is required.

Osmotic stimulation is a strong trigger for the secretion of AVP and copeptin. When copeptin was measured upon hypertonic (3%) saline infusion aiming at a serum sodium level of ≥ 149 mmol/L, a copeptin value of ≤ 4.9 mmol/L reliably distinguished patients with AVP-D from patients with PP with a diagnostic accuracy of 97% (7, 11) (Fig. 1). Importantly, prerequisites for the test include good venous access and near patient monitoring of serum sodium levels every 30 minutes to avoid too great an increase in plasma osmolality. In some patients the test should be avoided, including in those with heart failure and conditions where shifts in plasma osmolality may be deleterious, such as in those with seizure predisposition.

In order to overcome these limitations, arginine was proposed as an alternative and simpler secretagogue. Not only is arginine a stimulator of anterior pituitary secretion (25), it also stimulates copeptin secretion (24). Upon arginine infusion, copeptin measured 60 minutes after start of the infusion, using the cutoff of 3.8 pmol/L, had the highest diagnostic accuracy to differentiate between patients with AVP-D and PP patients (24). The most common adverse effect was mild nausea.

In a recent head-to-head trial comparing the diagnostic accuracy of hypertonic saline and arginine-stimulated copeptin, hypertonic saline stimulation proved to be superior to the arginine stimulation test with a diagnostic accuracy of 95.6% at a copeptin cutoff of 4.9 pmol/L vs 74.4% (with a cutoff of 3.8 pmol/L upon arginine). Nevertheless, an arginine-stimulated copeptin < 3.0 pmol/L or > 5.2 pmol/L diagnosed more than half of the patients with AVP-D and PP, respectively, with a specificity of > 90%. Overall, 72% of patients preferred the simpler arginine stimulation to hypertonic saline stimulation, as adverse effects were less frequent and milder (11), but a drawback is that arginine is not widely available.

Glucagon was also shown to stimulate copeptin with high diagnostic accuracy for the differentiation of AVP-D from PP in a pilot study, but larger studies are needed (26). Macimorelin does not increase copeptin levels (27).

### Use of Water Deprivation Tests

The indirect water deprivation test was first described in 1970 and remained the diagnostic gold standard for many decades (5). This test relies on the concept that the concentration of urine over a 16-hour period of fluid restriction is an indirect assessment of AVP secretion. In addition, the ability to concentrate urine in response to the synthetic V2 receptor-specific AVP analogue, desmopressin, is assessed at the end of the test. In patients where the urinary osmolality stays below 300 mOsm/kg during fluid deprivation but increases over 50% upon desmopressin administration the diagnosis of complete AVP-D can be made. Patients with a persistent low urinary osmolality and no response to desmopressin are diagnosed with AVP-R. Urinary concentration in patients with partial AVP and PP is expected to increase to levels above 300 mOsm/kg, while remaining below 800 mOsm/kg, with further increases in osmolality seen upon desmopressin injection of more than 9% in patients with partial AVP-D and less than 9% in patients with PP (5).

If reliable copeptin assays are not easily available, the water deprivation test remains a valid diagnostic test. A shorter version, that is, an overnight water deprivation test is often done as first diagnostic approach in clinical practice and is safe unless there is a high index of suspicion for complete AVP-D (in which case any restriction of fluid intake should be supervised). Published criteria to exclude AVP-D require morning fasted urine osmolality to be greater than 800 mOsm/kg. However, in practice, levels > 600 mOsm/kg generally exclude clinically significant AVP-D.

It is, however, important to be aware of the limitations in interpretation of the classically described test. First, the osmolality cutoffs described above were derived from a single study involving only 36 patients with post hoc assessment that has never been prospectively validated (5, 28). Second, the test results can be misleading in PP patients with a reduced renal medullary concentration gradient or partial AVP-R patients with sensitive response to desmopressin administration. Accordingly, 2 prospective studies evaluating the diagnostic accuracy of the indirect water deprivation test found it to be only 70% to 77%, with especially low accuracy in the differentiation between partial AVP-D and PP patients (7, 28). Finally, a large multicenter international study showed that two-thirds of the patients with PP who underwent a water deprivation test concentrated urine above 10% after desmopressin administration, leading to misclassification (10).

### Use of Therapeutic Trial in Certain Cases—Who, How, and What to Monitor

In view of past difficulties with performing and interpreting water deprivation tests, clinicians sometimes utilize a clinical trial of desmopressin to determine whether a patient has AVP-D. In some cases (eg, following neurosurgery of the sellar/suprasellar area, head trauma, or with diseases known to be associated with AVP-D, such as histiocytosis, sarcoidosis, germinomas, or other CNS etiologies), this is appropriate, since other etiologies of hypotonic polyuria are unlikely (as in our third case). However, with nontraumatic hypotonic polyuria in patients without known CNS disorders, this approach is suboptimal. If a patient with PP is treated with desmopressin and high levels of fluid intake continues, the resultant water retention can lead to dangerous hyponatremia. Conversely, if the polyuria does not decrease with desmopressin, this does not differentiate AVP-R from other forms of polyuria due to solute diuresis. Consequently, in such patients, confirmation of an accurate diagnosis via the tests described in this article is preferable before initiation of therapy with desmopressin. If a therapeutic trial of desmopressin is initiated, determination of a positive result should be done via measurement of increases in urine osmolality over short periods of time (24-72 hours) rather than weeks. Although there are no formal criteria to define a positive response to a therapeutic trial of desmopressin, use of the same criteria for determination of a positive response to desmopressin at the end of an indirect water deprivation test, namely an increase in urine osmolality of > 50%, is reasonable. It should be remembered that because of a combination of wash-out of the medullary concentration gradient and decreased synthesis of aquaporin-2 water channels in the absence of AVP stimulation of renal AVP V2 receptors, maximal urine concentration may not occur until after several days of desmopressin administration. If desmopressin is administered as a clinical trial in the absence of a confirmed diagnosis of AVP-D, it is essential that the serum sodium levels be checked within 2 to 5 days of the start of desmopressin to detect the development of hyponatremia before the patient becomes neurologically symptomatic. In the clinical trials of low-dose desmopressin to treat nocturia, hyponatremia occurred in a small number of individuals as early as 5 to 7 days after initiation of a single dose of desmopressin at night. Those at highest risk of the development of hyponatremia are elderly individuals, because desmopressin is cleared by the kidneys and the age-related decrease in glomerular filtration rate results in a longer half-life of desmopressin in older individuals, and females, likely because of increased expression of AVP V2 receptor mRNA and protein in females because of the location of the AVP V2 receptor gene on the X-chromosome.

### Problems, Approaches, and Solutions With Disorders of Thirst Sense

In normal physiology, thirst is stimulated in a linear fashion with increasing plasma osmolalities above 280 mOsm/kg (29).Patients with AVP-D and an intact thirst sense manage well when on desmopressin by drinking to thirst, usually maintaining their serum sodium the normal range. In some patients with AVP-D, the thirst sense is not fully normal (dysdipsic) or occasionally absent (adipsic), making water balance and maintenance of normonatremia more challenging. Disorders of thirst regulation are more likely with larger destructive hypothalamic lesions and pathology (for example craniopharyngioma) that destroy the hypothalamic osmoreceptors located in the anterior wall of the hypothalamus.

Assessing thirst is a crucial part of the overall assessment of the patient with hypotonic polyuria. This is most effectively done using a visual analogue diagram with the patient rating their thirst on a 1 (least) to 10 (most) thirst scale. If thirst sense is not in the high range when plasma osmolality is elevated (for example at the end of a hypertonic saline infusion or if fasting plasma osmolality is elevated), the patient has dysregulated thirst. Knowledge of this is important, as greater levels of patient education are needed about the volume of fluid intake, allowing for environmental factors such as heat and altitude. Such an approach is exemplified by the patient with true adipsic AVP-D, which can be one of the most challenging of all conditions in clinical endocrinology to manage (30). In this situation, patients need to take a fixed dose of desmopressin and a fixed volume of water/fluid per day defined by their weight when plasma osmolality and sodium are normal. The patient then weighs themselves once or twice a day, and the fluid volume is adjusted over the ensuing 24 hours—increased or decreased by the same volume in liters as the weight change in kilograms. Regardless of weight maintenance, any change in neurocognitive function necessitates measuring the serum sodium concentration to avoid both hyponatremia and hypernatremia. An alternative is frequent point-of-care testing for serum sodium concentration, where this is available and affordable.

## Conclusions

The last decade has seen a remarkable growth in our understanding of disorders of vasopressin-related polyuria. As the cases presented in this review clearly illustrate, we now have a more appropriate terminology for these disorders that more accurately reflects the underlying pathophysiology, but also better protects our patients from therapeutic misadventures by those who confuse these disorders with diabetes mellitus. However, these cases also illustrate that we now have the ability to more accurately diagnose among the various causes of hypotonic polyuria using newly developed assays for the C-terminal fragment of the AVP prohormone, copeptin. This should be no surprise to endocrinologists, who have long been using a similar fragment of the insulin prohormone, C-peptide, as a surrogate marker of insulin secretion. As always, there remains more to be understood about the disorders causing AVP-D, AVP-R, and PP, as well as the most effective therapies for these disorders, but we are now on more solid ground to accomplish this based on the advances described in this practical review of how clinicians should approach patients with suspected hypotonic polyuria. Perhaps most importantly, by embracing these new pathophysiologically descriptive and diagnostic concepts, our endocrinology trainees will be better equipped to diagnose and treat disorders of vasopressin-related polyuria than we were.

## Data Availability

Data sharing is not applicable to this article as no datasets were generated or analyzed during the current study

## Abbreviations

AVP-D: arginine vasopressin deficiency
AVP-R: arginine vasopressin resistance
CNS: central nervous system
eGFR: estimated glomerular filtration rate
HbA1c: glycated hemoglobin
ILA: immunoluminometric assay
MRI: magnetic resonance imaging
PP: primary polydipsia
T4: thyroxine
TSH: thyrotropin (thyroid stimulating hormone)

## References

[1] Christ-Crain M, Bichet DG, Fenske WK, et al Diabetes insipidus. Nat Rev Dis Primers. 2019;5(1):54. 31395885 10.1038/s41572-019-0103-2

[2] Arima H, Cheetham T, Christ-Crain M, et al Changing the name of diabetes insipidus: a position statement of the working group for renaming diabetes insipidus. J Clin Endocrinol Metab. 2022;108(1):1‐3. 36355385 10.1210/clinem/dgac547 PMC9759163

[3] Atila C, Loughrey PB, Garrahy A, et al Central diabetes insipidus from a patient's perspective: management, psychological co-morbidities, and renaming of the condition: results from an international web-based survey. Lancet Diabetes Endocrinol. 2022;10(10):700‐709. 36007536 10.1016/S2213-8587(22)00219-4

[4] SONMED International . https://www.snomed.org/news/snomed-responds-to-community-call-for-improved-diabetes-insipidus-terminology-in-snomed-ct#:∼:text=In%20October%202022%2C%20the%20Working, of%20this%20set%20of%20conditions.

[5] Miller M, Dalakos T, Moses AM, Fellerman H, Streeten DH. Recognition of partial defects in antidiuretic hormone secretion. Ann Intern Med. 1970;73(5):721‐729. 5476203 10.7326/0003-4819-73-5-721

[6] Timper K, Fenske W, Kühn F, et al Diagnostic accuracy of copeptin in the differential diagnosis of the polyuria-polydipsia syndrome: a prospective multicenter study. J Clin Endocrinol Metab. 2015;100(6):2268‐2274. 25768671 10.1210/jc.2014-4507

[7] Fenske W, Refardt J, Chifu I, et al A copeptin-based approach in the diagnosis of diabetes insipidus. N Engl J Med. 2018;379(5):428‐439. 30067922 10.1056/NEJMoa1803760

[8] Snitow ME, Bhansali RS, Klein PS. Lithium and therapeutic targeting of GSK-3. Cells. 2021;10(2):255. 33525562 10.3390/cells10020255 PMC7910927

[9] Atila C, Beck J, Erlic Z, et al Psychopathological characteristics in patients with arginine vasopressin deficiency (central diabetes insipidus) and primary polydipsia compared to healthy controls. Eur J Endocrinol. 2024;190(5):354‐362. 38551325 10.1093/ejendo/lvae040

[10] Fenske W, Quinkler M, Lorenz D, et al Copeptin in the differential diagnosis of the polydipsia-polyuria syndrome–revisiting the direct and indirect water deprivation tests. J Clin Endocrinol Metab. 2011;96(5):1506‐1515. 21367924 10.1210/jc.2010-2345

[11] Refardt J, Atila C, Chifu I, et al Arginine vs hypertonic saline-stimulated copeptin to diagnose AVP deficiency. N Engl J Med. 2023;389(20):1877‐1887. 37966286 10.1056/NEJMoa2306263

[12] Land H, Schütz G, Schmale H, Richter D. Nucleotide sequence of cloned cDNA encoding bovine arginine vasopressin-neurophysin II precursor. Nature. 1982;295(5847):299‐303. 6276766 10.1038/295299a0

[13] Levy B, Chauvet MT, Chauvet J, Acher R. Ontogeny of bovine neurohypophysial hormone precursors. II. Foetal copeptin, the third domain of the vasopressin precursor. Int J Pept Protein Res. 1986;27(3):320‐324. 3710692

[14] Robertson GL . The regulation of vasopressin function in health and disease. Recent Prog Horm Res. 1976;33:333‐385. 801194 10.1016/b978-0-12-571133-3.50015-5

[15] Zerbe RL, Robertson GL. A comparison of plasma vasopressin measurements with a standard indirect test in the differential diagnosis of polyuria. N Engl J Med. 1981;305(26):1539‐1546. 7311993 10.1056/NEJM198112243052601

[16] Wun T . Vasopressin and platelets: a concise review. Platelets. 1997;8(1):15‐22. 16793628 10.1080/09537109777492

[17] Preibisz JJ, Sealey JE, Laragh JH, Cody RJ, Weksler BB. Plasma and platelet vasopressin in essential hypertension and congestive heart failure. Hypertension. 1983;5(2 Pt 2):I129‐I138. 6826223 10.1161/01.hyp.5.2_pt_2.i129

[18] Jane Ellis M, Livesey JH, Evans MJ. Hormone stability in human whole blood. Clin Biochem. 2003;36(2):109‐112. 12633759 10.1016/s0009-9120(02)00440-x

[19] Takagi H, Hagiwara D, Handa T, et al Diagnosis of central diabetes insipidus using a vasopressin radioimmunoassay during hypertonic saline infusion. Endocr J. 2020;67(3):267‐274. 31748430 10.1507/endocrj.EJ19-0224

[20] Balanescu S, Kopp P, Gaskill MB, Morgenthaler NG, Schindler C, Rutishauser J. Correlation of plasma copeptin and vasopressin concentrations in hypo-, iso-, and hyperosmolar states. J Clin Endocrinol Metab. 2011;96(4):1046‐1052. 21289257 10.1210/jc.2010-2499

[21] Fenske WK, Schnyder I, Koch G, et al Release and decay kinetics of copeptin vs AVP in response to osmotic alterations in healthy volunteers. J Clin Endocrinol Metab. 2018;103(2):505‐513. 29267966 10.1210/jc.2017-01891

[22] Morgenthaler NG, Struck J, Alonso C, Bergmann A. Assay for the measurement of copeptin, a stable peptide derived from the precursor of vasopressin. Clin Chem. 2006;52(1):112‐119. 16269513 10.1373/clinchem.2005.060038

[23] Sailer CO, Refardt J, Blum CA, et al Validity of different copeptin assays in the differential diagnosis of the polyuria-polydipsia syndrome. Sci Rep. 2021;11(1):10104. 33980941 10.1038/s41598-021-89505-9 PMC8114908

[24] Winzeler B, Cesana-Nigro N, Refardt J, et al Arginine-stimulated copeptin measurements in the differential diagnosis of diabetes insipidus: a prospective diagnostic study. Lancet. 2019;394(10198):587‐595. 31303316 10.1016/S0140-6736(19)31255-3

[25] Merimee TJ, Rabinowtitz D, Fineberg SE. Arginine-initiated release of human growth hormone. Factors modifying the response in normal man. N Engl J Med. 1969;280(26):1434‐1438. 5786514 10.1056/NEJM196906262802603

[26] Atila C, Gaisl O, Vogt DR, Werlen L, Szinnai G, Christ-Crain M. Glucagon-stimulated copeptin measurements in the differential diagnosis of diabetes insipidus: a double-blind, randomized, placebo-controlled study. Eur J Endocrinol. 2022;187(1):65‐74. 35521789 10.1530/EJE-22-0033

[27] Urwyler SA, Lustenberger S, Drummond JR, et al Effects of oral macimorelin on copeptin and anterior pituitary hormones in healthy volunteers. Pituitary. 2021;24(4):555‐563. 33615399 10.1007/s11102-021-01132-9 PMC8270818

[28] Fenske W, Allolio B. Clinical review: current state and future perspectives in the diagnosis of diabetes insipidus: a clinical review. J Clin Endocrinol Metab. 2012;97(10):3426‐3437. 22855338 10.1210/jc.2012-1981

[29] Leib DE, Zimmerman CA, Zachary AK. Thirst. Curr Biol. 2016;26(24):R1260‐R1265. 27997832 10.1016/j.cub.2016.11.019 PMC5957508

[30] Cuesta M, Hannon MJ, Thompson CJ. Adipsic diabetes insipidus in adult patients. Pituitary. 2017;20(3):372‐380. 28074401 10.1007/s11102-016-0784-4

